# Transcriptomic and proteomic analysis reveals wall-associated and glucan-degrading proteins with potential roles in *Phytophthora infestans* sexual spore development

**DOI:** 10.1371/journal.pone.0198186

**Published:** 2018-06-13

**Authors:** Xiaofan Niu, Audrey M. V. Ah-Fong, Lilianna A. Lopez, Howard S. Judelson

**Affiliations:** Department of Microbiology and Plant Pathology, University of California, Riverside, CA, United States of America; Tallinn University of Technology, ESTONIA

## Abstract

Sexual reproduction remains an understudied feature of oomycete biology. To expand our knowledge of this process, we used RNA-seq and quantitative proteomics to examine matings in *Phytophthora infestans*. Exhibiting significant changes in mRNA abundance in three matings between different A1 and A2 strains compared to nonmating controls were 1170 genes, most being mating-induced. Rising by >10-fold in at least one cross were 455 genes, and 182 in all three crosses. Most genes had elevated expression in a self-fertile strain. Many mating-induced genes were associated with cell wall biosynthesis, which may relate to forming the thick-walled sexual spore (oospore). Several gene families were induced during mating including one encoding histidine, serine, and tyrosine-rich putative wall proteins, and another encoding prolyl hydroxylases which may strengthen the extracellular matrix. The sizes of these families vary >10-fold between *Phytophthora* species and one exhibits concerted evolution, highlighting two features of genome dynamics within the genus. Proteomic analyses of mature oospores and nonmating hyphae using isobaric tags for quantification identified 835 shared proteins, with 5% showing >2-fold changes in abundance between the tissues. Enriched in oospores were β-glucanases potentially involved in digesting the oospore wall during germination. Despite being dormant, oospores contained a mostly normal complement of proteins required for core cellular functions. The RNA-seq data generated here and in prior studies were used to identify new housekeeping controls for gene expression studies that are more stable than existing normalization standards. We also observed >2-fold variation in the fraction of polyA^+^ RNA between life stages, which should be considered when quantifying transcripts and may also be relevant to understanding translational control during development.

## Introduction

Sexual reproduction contributes to the survival of many species. In the filamentous microbial eukaryotes known as oomycetes, sexual development culminates in the formation of oospores. These are made both by homothallic (self-fertile) species and in pairings of the two mating types of heterothallics, which are called A1 and A2 in *Phytophthora* [1]. Oospores have thick walls that allow them to survive harsh treatments including chemical fumigation, freezing, and microbial attack. Oospores can remain viable in soil or plant debris for years [2]. Consequently, oospores serve as inoculum at the start of the growing season in many pathosystems involving oomycetes such as *Phytophthora*, *Pythium*, and downy mildews [3–5]. The sexual cycle also contributes to fitness by yielding new genotypes. This is well-described in the potato late blight system, where recombination between strains of the pathogen, *Phytophthora infestans*, has led to more aggressive genotypes [6, 7].

Our understanding of the biology of mating in oomycetes is limited. In *Phytophthora*, mating hormones have been identified but their effects on gene expression are undescribed [8]. One challenge to studying oospores is that they comprise a small fraction of culture biomass, and are not separated easily from hyphae. Oospores are formed when male and female gametangia (antheridia and oogonia) fuse, but these sexual structures stay attached to and embedded within the vegetative thallus. An additional challenge is the difficulty of breaking oospore walls, which contain multiple layers of β-glucans [9]. Nevertheless, micrographic and limited biochemical studies indicated that oospores contain substantial lipid reserves in addition to the carbon stored in its wall [10]. Ribosomes, mitochondria, and proteins in the electron transport chain have been described as being absent or degraded [11, 12]. The metabolic capabilities of oospores are still largely unknown, although some enzymatic activities are known to be present based on histochemical staining.

In 2006, we reported the results of a microarray study of *P*. *infestans* that identified 87 unigenes induced >10-fold during mating [13]. Limitations of that project became evident after the genome sequence was released in 2009 [14]. For example, the microarray was based largely on expressed sequence tags (ESTs), which were found to correspond to only about two-thirds of the 17,797 currently predicted genes of *P*. *infestans*. Moreover, multiple unigenes represented on the array were discovered to correspond to the same gene, many 'genes' were in fact transposable elements, and the fragmentary nature of ESTs hampered functional annotation. The dynamic range of microarray technology also restricted the sensitivity and accuracy of the results. These issues reflect the limitations of the methodologies available at the time, and are not unique to *P*. *infestans*.

The main goal of the current study is to advance our understanding of oomycete sexual development, using *P*. *infestans* as a model. The use of RNA-seq with three different crosses resulted in the discovery of more than 1000 mating-induced genes including 455 that were upregulated by >10-fold. A quantitative proteomics analysis of mature oospores and vegetative hyphae revealed broad similarities between the tissues, except for an enrichment in oospores of enzymes such as β-glucanases that may aid germination. As a secondary goal of this study, we describe using the RNA-seq data to identify improved housekeeping controls for gene expression studies.

## Materials and methods

### Growth and mating conditions

*P*. *infestans* strains of the A1 mating type were 8811 (from the United Kingdom), 88069 (The Netherlands), and 1306 and R0 (United States). A2 isolates were 550 and 618 (Mexico), and E13 (Egypt). Self-fertile strain 6.11 was a sexual offspring of strains 2411 and 510 [15]. Nonmating cultures were grown in the dark at 18°C on rye A media containing 1.5% agar [16]. Mating cultures were established by placing parallel strips of inoculum on rye agar plates, separated by about 2 cm. Oospore viability was measured by placing a 4 mm^2^ piece of mating tissue in 30 μl of 0.1% tetrazolium bromide (MTT) in 0.1 M potassium phosphate pH 5.8, followed by incubation of the mixture at 37ºC for 2 days [17]. Matings for RNA or protein analysis were performed by placing a 0.4 μm pore polycarbonate filter (Sterlitech) on the rye agar plate prior to inoculation.

### RNA-seq analysis

RNA was obtained by grinding tissue in liquid nitrogen, followed by extraction using the Plant Total RNA kit from Sigma. After verifying RNA quality using an Agilent 2100 Bioanalyzer, indexed stranded libraries were prepared using the Illumina Truseq kit. The libraries were quantitated using a Qubit 2.0 fluorescence reader, multiplexed, and sequenced on a NextSeq500 to obtain 75-nt single-end reads. Reads passing the quality filter were aligned to the *P*. *infestans* reference genome [14] using Bowtie 2.2.5 and Tophat 2.0.14, allowing for one mismatch. Expression levels and differential expression calls were obtained using edgeR with TMM normalization, a generalized linear model, and Benjamini-Hochberg false discovery rate (FDR) calculations [18]. GO term enrichment analysis was performed using GoStat, with *P* values reported as FDR values [19]. Fastq files for RNA-seq analysis are deposited in the NCBI database as Bioproject PRJNA445478.

### Sequence analysis

Predictions of subcellular targeting were obtained using the TargetP and Mitofates programs [20, 21]. Proteins were considered to be mitochondrial only if predicted by both approaches. Orthologs of M96 and prolyl hydroxylase genes in *Phytophthora* spp. were identified by searching the Fungidb.org database using BlastP with an *E*-value threshold of 10^-20^, without the low-complexity filter. A threshold of 10^−10^ was used when searching *Pythium* genomes. M96 sequences from *P*. *mirabilis* and *P*. *phaseoli* were obtained by PCR using degenerate primers based on *P*. *infestans* sequences [22]. Phylogenetic analyses were performed with protein alignments generated by MUSCLE [23], which were trimmed using TCS [24]. Trees were then made using PhyML [25] with the LG substitution model, with branch support obtained using the SH-like aLRT option. Trees were also generated using MrBayes 3.6 [26] with 400,000 generations, sampling every 200 cycles, 10,000 burn-in cycles, gamma distributed variation, and four heated chains. The topologies of trees developed using PhyML and MrBayes closely resembled each other.

Evaluations of ortholog copy number based on read depth used Illumina libraries deposited at the NCBI Short Read Archive for each species (accessions SRX027285, SRX1116285, SRX1953149, SRR1046800, SRX889636, SRX1117110, SRX2200946, SRX038792, SRX1374272, SRX156070, SRX1374272, SRX069904, SRX020087). Each library was searched using BLASTN with DNA sequences taken from the 5' and 3' ends of representative M96 or prolyl hydroxylase genes, along with sequences of equal length from the 5' and 3' ends of five single-copy genes. Copy numbers were calculated based on the number of hits compared to the single-copy controls.

### Oospore purification

Matings were performed on a polycarbonate membrane. After 30 days, 1 × 9 cm strips of each mating zone were cut with a scalpel and peeled off of the membrane. Both enzyme-based and mechanical methods for separating oospores from hyphae were tested. In tests of enzymes, tissues pooled from ten or more plates were homogenized five times for 2 min each in 50 ml of sterile water at 4°C in a Brinkman Polytron (speed 7), passed through 100 μm nylon mesh, and concentrated by centrifugation for 10 min at 4,000× *g*. Oospores and hyphae in the filtrate were then resuspended in 20 ml of water, and washed three to five times in water by spinning at 650 × *g* for 5 min with a final spin of 4,000 × *g* for 10 min. The pellet was resuspended in 20 mg/ml of Novozyme 234 (Calbiochem) or Glucanex (Sigma) with gentle shaking for 12 hr. After enzyme treatment, the oospores were washed five to eight times with water containing 0.1% sodium dodecyl sulfate (SDS) using 10 min spins at 4,000 × *g*. A mechanical purification method was employed for protein sequencing studies to avoid signals resulting from residual enzyme. Strips of mating tissue were combined with acid-washed glass beads (50 μm diameter) in water, and homogenized for 1.5 min in a Bio101/Savant Fastprep FP120 homogenizer at speed 6. Two 1 × 9 cm strips were used per tube with 100 μl of beads and 1.5 ml of water. For large-scale preparations, 200 strips were distributed among 100 tubes. After agitation, the mixture was passed through 100 μm and then 35 μm nylon mesh. The flow-through was centrifuged at 5,700 × *g* for 5 min, and then the pellet was resuspended in an equal volume of water, centrifuged at 4,000 × *g*, and the supernatant discarded. This wash was repeated three times, with two final 5 min washes at 650 × *g*. About 2 × 10^7^ oospores were obtained from 200 mating zone strips.

### Extraction of proteins

Proteins were extracted from oospores or nonsporulating hyphae (the latter grown for three days in rye broth) by placing the tissue in 20 mM Tris-HCl, pH 8.0, 150 mM NaCl, 10 mM EDTA, pH 8.0, 0.2% Nonidet P-40, 0.02 mg/ml heparin, 1.5 mM DTT, 1 mM PMSF, and 20 units/ml DNase I. This was agitated twice for 1 min at speed 6 in a Qbiogene FastPrep beadbeater with 1/4 inch ceramic spheres and 200 μm glass beads, and centrifuged at 20,000 x *g* for 10 min to remove debris. Protein concentrations were determined using the Non-interfering Protein Assay Kit from EMD Millipore. We also tried performing extractions from the debris using the same buffer with varying concentrations of SDS, but measurable amounts of protein were not obtained.

### Peptide isolation and labeling with isobaric tags

Protein samples (100 μg) were separated by 10% acrylamide SDS-PAGE until the samples had moved about 1.75 cm into the separating gel, and then stained with colloidal blue. Regions containing protein were excised, cut into 1 mm^2^ fragments, submerged in 100 μl of 25 mM ammonium bicarbonate in 50% acetonitrile in 1.5 ml tubes, and vortexed for 1 hr. The buffer was replaced by fresh buffer, and the treatment repeated until visible dye was removed. The pieces were then incubated in 200 μl acetonitrile for 15 min at room temperature, and dried using a centrifugal vacuum concentrator for 1 hr. To each sample we then added 30 μl of 0.5 M triethylammonium bicarbonate pH 8.5 plus 1 μl of 2% SDS. After vortexing, tris-(2-carboxyethyl)-phosphine was added to 5 mM, followed by incubation at 60°C for 1 hr. The buffer was removed, replaced with enough 12.5 ng/μl trypsin plus 50 mM ammonium bicarbonate pH 7.8 to cover the gel pieces, and incubated overnight at 37°C. The liquid was then replaced with 50 μl of 5% acetonitrile and 0.1% trifluoroacetic acid (TFA), and the tube vortexed for 15 min. The supernatant was removed and the treatment repeated three times, each time adding an additional 50 μl of 5% acetonitrile and 0.1% TFA. All supernatants were then pooled and reduced to 10 μl using a centrifugal vacuum concentrator.

To tag the peptides with 4-plex iTRAQ reagent (Isobaric Tags for Relative and Absolute Quantitation; Applied Biosystems), 100 μg of peptides were mixed with the reagent and incubated at room temperature for 1 hr. Proteins from A1 hyphae were labeled with the iTRAQ 114 reporter ion, A2 hyphae with reporter ion 116, and two replicate oospore samples with 115 and 117 reporter ions, respectively. The reactions were then pooled and passed through a cation-exchange cartridge.

### Protein mass spectrometry

Analyses were performed in the W.M. Keck Proteomics Facility of the University of California, Riverside using MudPIT (Multi-Dimensional Protein Identification Technology [27]. This employed a Waters nanoACQUITY UPLC with cation exchange chromatography (SCX), and a Waters Q-TOF nano-ESI mass spectrometer. Samples were divided into 10 fractions and analyzed in four runs. Spectra were analyzed with MASCOT (Matrix Science) and searched against the *P*. *infestans* protein database. Searches assumed a tryptic digest, one peptide with 95% confidence, up to one missed cleavage per peptide, carboxyamidomethylation, 4-plex iTRAQ on N-termini and lysines as fixed mass modifications, and oxidation (*M*) as a variable mass modification. Monoisotopic mass values were used, with peptide mass tolerance and fragment mass tolerance set at 60 ppm and 0.2 Da, respectively, and a cut-off value MASCOT score of 50. To perform quantitative analyses, signature peak values of iTRAQ reporter ions were extracted from the Pkl file generated by MASCOT, using mass-to-charge values (*m/z*) of 114.1±0.01, 115.1±0.01, 116.1±0.01, or 117.1±0.01 for each reporter ion. Relative expression was calculated by averaging the signature peak values of the reporter ions of all unique peptides belonging to a protein. Abundance ratios were determined after median normalization, and *p*-values assigned to fold-change values using Bayesian statistics, using the Cyber-T program with a confidence value of 6 [28]. Bayesian estimation was used to measure the significance of differences between functional groups [29]. Several samples were also subjected to label-free analysis, and proteins quantified by the emPAI method [30].

### Reverse transcription-quantitative polymerase chain reaction (RT-qPCR)

Candidate target genes were selected based on the stability of their CPM (counts per million) values in nonsporulating hyphae grown in pea broth, rye broth, rye agar, amino acid-based minimal media [31], ammonium sulfate-based minimal media [32], sporulating hyphae on rye agar, 10-day matings on rye agar, hyphae treated with metalaxyl in rye broth (6 hr at IC_10_), sporangia, sporangia chilled to stimulate zoosporogenesis, swimming zoospores, and germinated cysts forming appressoria [33], and infected potato tubers and tomato leaves at early, middle, and late stages of infection [34, 35]. Primers (S1 Table) were designed to amplify bands of about 150 bp from the 3’ end of the target mRNA. cDNA was prepared using the Maxima First-Strand RT-PCR Kit (Thermo) with DNAse-treated RNA. Gel electrophoresis was used to confirm the absence of bands in samples lacking reverse transcriptase. RT-qPCR was performed in triplicate using 15 to 18 ng of cDNA (depending on experiment), a CFX Connect instrument (Biorad), and the DyNAmo HS SYBR Green qPCR kit (Thermo). Program parameters were 95°C for 15 min, followed by 40 three-step cycles consisting of 10 sec at 94°C, 20 sec at 55°C, and 15 sec at 72°C. The fidelity of amplification was confirmed using melt curves. A dilution series of template was used to calculate primer efficiencies, which ranged from 89 to 93%. For analyses of mRNA in plants, calculations included an adjustment to account for the fraction of mRNA from *P*. *infestans* based on RNA-seq analysis. The stability of each gene was determined using NormFinder, geNorm, delta Ct, Bestkeeper, and RefFinder [36–39].

### Purification of polyadenylated RNA

Paramagnetic oligo d(T)_25_ beads (New England Biolabs) were washed twice with 100 μl of RNA binding buffer (20 mM Tris-HCl pH 7.5, 1 M LiCl, 2 mM EDTA) using a magnetic separator, prior to the addition of RNA, which had been quantified using the Qubit Broad-Range Assay kit (Thermo). To ensure that all samples were treated similarly, the following heating and cooling steps were performed using programs in a thermocycler. Five micrograms of RNA in 50 μl of binding buffer were mixed with an equal volume of beads at room temperature, heated at 65°C for 5 min, cooled to 4°C, and incubated at room temperature (22°C) for 10 min. The beads were then washed twice using 200 μl of washing buffer (10 mM Tris-HCl pH 7.5, 0.15 M LiCl, 1 mM EDTA) to remove most non-polyadenylated RNAs. The remainder was removed by a second round of binding. This involved incubating the beads with 50 μl of 10 mM Tris-HCl pH 7.5 at 80°C for 2 min in the thermocycler, followed by a shift to 25°C. An additional 50 μl of RNA binding buffer was then added. After 5 min at room temperature (22°C), the beads were washed once with 200 μl of washing buffer, and then 17 μl of 10 mM Tris-HCl pH 7.5 was added. The beads were then incubated at 80°C for 2 min to elute the polyA^+^ fraction. The temperature was then dropped to 25°C, the tube was placed immediately on the magnetic stand, and the eluent was transferred to a fresh tube. The amount of RNA was quantified using the Qubit RNA HS Kit and analyzed using an Agilent 2100 Bioanalyzer to confirm the removal of ribosomal RNA.

## Results

### Selection of parents for analysis

**Fig 1:**
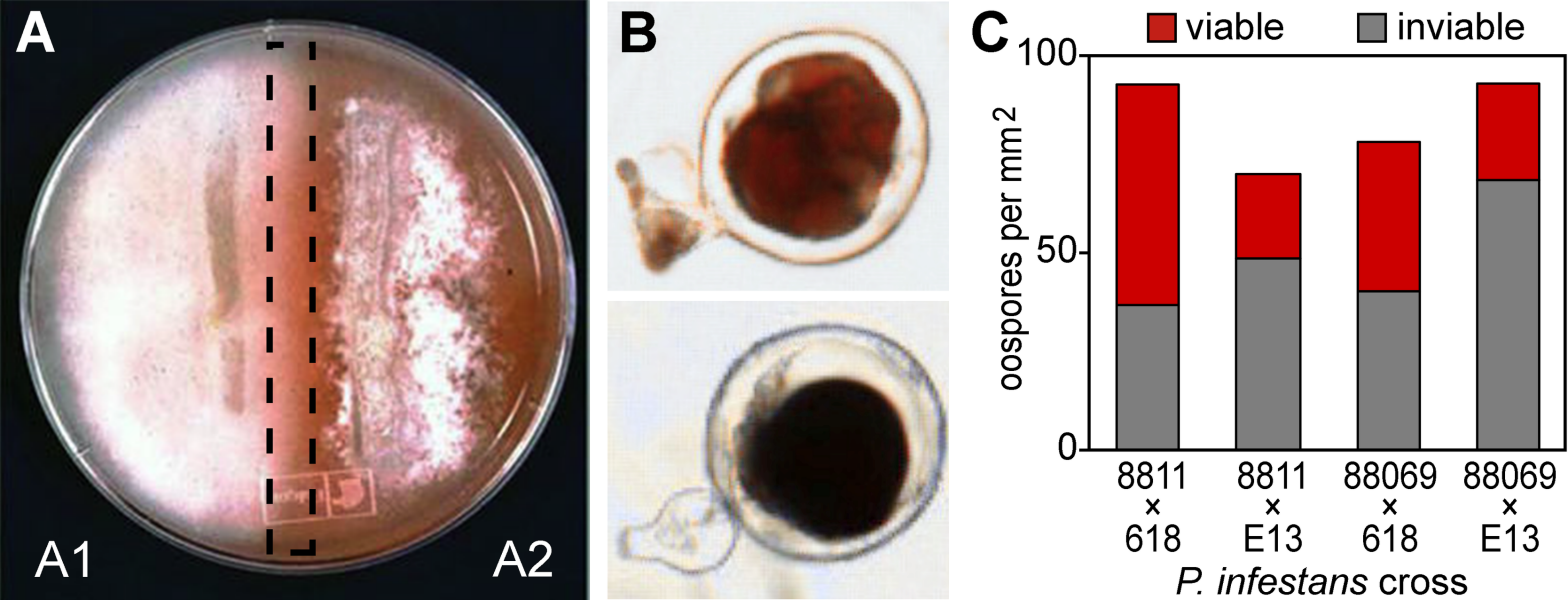
Matings in *P*. *infestans*. (**A)** mating initiated using parallel strips of A1 and A2 inoculum. The zone excised for isolating RNA or protein is denoted by the dashed box. For obtaining RNA or protein, a polycarbonate membrane was placed on top of the agar prior to inoculation, in order to facilitate tissue recovery. An orange disk of paper was placed under the plate to increase contrast in the photograph. (**B)** MTT assay showing red-stained viable (top) and inviable black-stained (bottom) oospores (bottom). Oospores are typically about 32 μm in diameter. **(C)** oospore production in pairings of strains 8811 or 88069 (A1) with strains 618 or E13 (A2). Graphed are the density of oospores and the fraction of oospores that were determined to be viable (red) or inviable (black). The image in panel A is taken from a prior publication [1].

Four A1 and three A2 strains of *P*. *infestans* were mated in all combinations in order to identify pairings that yielded well-defined mating zones, in which oospore production is maximized and sporulating aerial hyphae is minimized. All interactions yielded oospores, but mating zones were most clearly delimited in pairings of A1 strains 8811 or 88069 and A2 strains 618 or E13. An example is shown in Fig 1A, in which the mating zone contained few aerial hyphae or asexual sporangia, with oospores formed beneath the agar surface.

Tests using the tetrazolium salt MTT indicated the presence of both viable and inviable oospores in each pairing (red and black-stained, respectively; Fig 1B). The variability of oospore viability in *Phytophthora* has been described previously [17]. The density of oospores and proportion of viable oospores in 8811 × 618, 8811 × E13, 88069 x 618, and 88069 × E13 pairings are shown in Fig 1C. The first three pairings were selected for further analysis since they produced the highest fraction of viable oospores.

### Identification of mating-induced genes

RNA-seq was used to compare the mating zones of 10-day mating cultures with 10-day cultures of the parents grown separately. After 10 days, mating cultures contain gametangia and oospores of varying levels of maturity. Two or three biological replicates were prepared for each of the three mating (A1 plus A2) and four non-mating treatments (A1 and A2 alone), representing a total of seven mating and eight non-mating samples. Illumina technology was used to obtain an average of 27 million 75-nt single-end reads per sample, of which about 89% mapped to the *P*. *infestans* genome (S2 Table). A total of 16,631 genes were defined as expressed based on having an average CPM (counts per million mapped reads) above 1 in at least one tissue sample.

**Fig 2:**
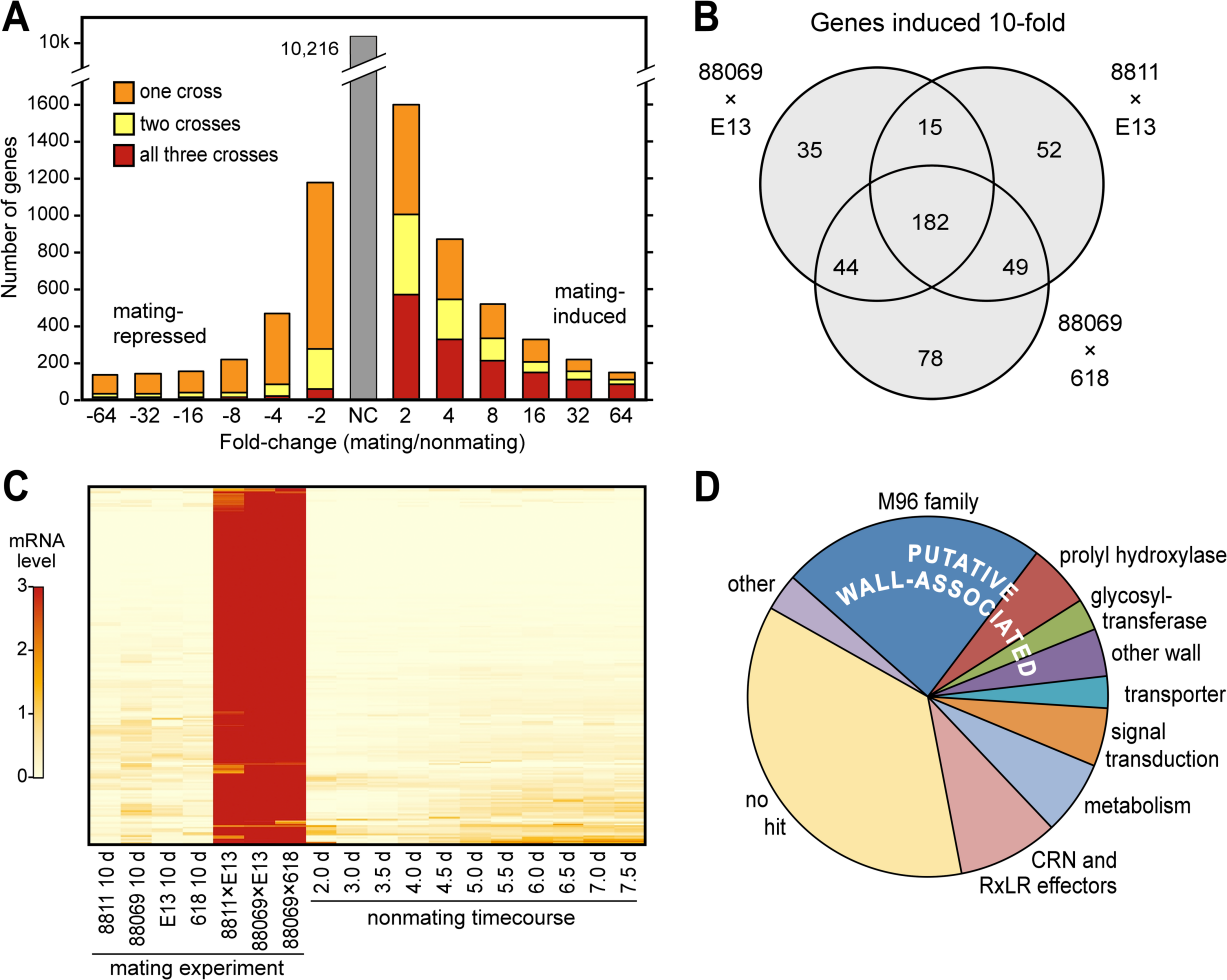
RNA-seq analysis of transcriptional changes in *P*. *infestans* during mating. **(A)** number of genes induced (right) or repressed (left) during mating compared to non-mating A1 and A2 controls. The bar labeled 4, for example, denotes the number of genes with a ≥4-fold increase. Color-coding indicates the number of genes changing in one (orange), two (yellow), or all three (red) crosses. **(B)** Venn diagram comparing genes showing >10-fold mating induction (FDR<0.05) in the three crosses. **(C)** Heatmap of genes showing >10-fold induction in at least one cross. Labeled as "mating experiment" are the 10-day nonmating and mating samples, which were used for the analyses in panel A. The samples labeled "nonmating timecourse" are from 2 to 7.5-day old cultures of an A1 isolate. **(D)** Classification of genes showing >10-fold induction in all three crosses, which are listed along with their predicted functions in S5 Table.

About 1170 genes showed significant changes between mating and non-mating cultures in all three crosses, based on an FDR threshold of 0.05 calculated using the Benjamini-Hochberg method. Within this group we observed 325 genes with a >4-fold increase in all three crosses compared to non-mating controls, 545 with this degree of increase in at least two crosses, and 873 in at least one cross (Fig 2A). Showing >10-fold up-regulation in at least one and all three crosses were 455 and 182 genes, respectively (Fig 2B). Relatively few genes were down-regulated in all three crosses. Expression levels, fold-change values, and FDR statistics of all *P*. *infestans* genes are shown in S3 and S4 Tables.

Our previous microarray study had identified 87 mating-induced unigenes [13]. The 87 corresponds to 74 protein-coding genes in the current annotation of the *P*. *infestans* genome. Of these 74 genes, 72 appeared as mating-induced in the RNA-seq data. Therefore, the present study raised the number of mating-induced genes by 5 to 10-fold, depending on the number of crosses being compared.

That not all genes were scored as induced in all three crosses was due usually to quantitative differences. This is shown in Fig 2C for the 455 genes that were upregulated by >10-fold in at least one cross. The data on the left side of the heatmap ("mating experiment") indicate that all genes were induced in each cross, albeit to varying levels. The variation was presumably due to differences in the intensity of oospore development.

Further support for the premise that the upregulated genes were authentically mating-induced comes from the samples on the right side of the heatmap in Fig 2C ("nonmating timecourse"), which portrays 2 to 7.5-day cultures of a single strain. These data are useful since genes calculated as being mating-induced might fall into two classes. The first includes genes that are expressed more during mating than at any time in a non-mating culture. The second class represents genes that are down-regulated in 10-day non-mating cultures, but have sustained expression in the 10-day mating cultures. The data indicate that >95% genes are truly mating-induced.

### Functions of mating-induced genes

Predicted functions of the 455 genes that were induced >10-fold during mating are shown in Fig 2D and S5 Table. About two-thirds of the genes encode proteins that are similar to sequences with annotated functions in GenBank, motifs in protein domain databases, or previously described families of oomycete genes. GO term enrichment analysis of the mating-induced genes identified two classes that were over-represented. These encoded sodium-dependent phosphate transmembrane transporters (GO:0015321; *Padj* = 0.01) and elicitin-like proteins (GO:0051704; *Padj* = 0.02). The latter GO term is defined as multiorganism process, but the primary role of elicitin-like proteins likely involves binding to sterols [40].

More than half of the proteins are predicted to have functions associated with cell walls. Most predominant is a gene family named M96 (*e*.*g*. PITG_05896; [22]). This family contains 49 annotated 'genes', including nine containing internal stop codons. Excluding the latter, their predicted proteins have signal peptides for secretion and a median mature size of 271 amino acids. All were induced >100-fold during mating. The N-terminal third of the proteins are histidine-rich (typically 14 mole-percent), with the remainder being high in glycine, serine and tyrosine (typically 50 mole-percent). These are known features of extracellular matrix proteins [41, 42]. A serine and tyrosine-rich protein with weak sequence similarity to M96 has also been localized to the oospore wall of *Py*. *oligandrum* [43].

The second largest group is a gene family that encodes proteins with a 2-oxoglutarate (2OG)-dependent dioxygenase domain, which is defined by Interpro domain IPR005123 and by Pfam family 13640. These proteins are also known as prolyl 4-hydroxylases. The family has 17 members in *P*. *infestans*, of which 12 were mating-induced (*e*.*g*. PITG_06187, induced >100-fold) although two of the genes appear truncated and lack the dioxygenase domain. Prolyl 4-hydroxylases increase the stability of collagen, which is the main extracellular structural protein of animals, and play a similar role in plant cell walls [44, 45]. Some members of the family have an oxygen-sensing role which may be independent of their function in the extracellular matrix [46].

Other mating-induced genes with potential roles in cell walls include six encoding glycosyltransferases (*e*.*g*. PITG_14139, >100-fold induced), one encoding a transglutaminase (PITG_13497, >100-fold induced), one encoding a tyrosinase (PITG_05533, 52-fold induced), and genes encoding the elicitin-like proteins mentioned earlier (*e*.*g*. PITG_02505, >100-fold induced). All of these predicted gene products bear signal peptides. Extracellular transglutaminases are believed to crosslink proteins in cell walls, while tyrosinases crosslink molecules in the wall [47]. In plants, similar glycosyltransferases are Golgi-localized and contribute to wall synthesis by assembling hemicellulose, other glucans, and glycoproteins [48]. They might also participate in forming laminarin, a β(1→3) glucan with β(1→6) branches that is used as a storage carbohydrate in oomycetes and brown algae [49].

Several mating-induced genes code for proteins with potential roles in signal transduction, including several protein phosphatases (*e*.*g*. PITG_05159 and PITG_22283, both 100-fold mating-induced) and transcriptional regulators. Two of the latter encode homeobox transcription factors PITG_01080 and PITG_01335, which averaged 29 and 60-fold induction during mating, respectively. Other transcriptional regulators included a family of five genes induced by about 20-fold (*e*.*g*. PITG_14403) that encode pirin, which is a non-heme Fe-containing protein that controls the NF-κB transcription factor in humans [50]. Also induced >10-fold was gene PITG_15600, which encodes an ortholog of Nipped-B, which is a regulator of transcription and sister chromatid cohesion in animals [51].

A surprising observation was the identification of seven mating-induced CRN-like proteins and 11 RxLRs among the genes induced by >10-fold in all three crosses (S5 Table). These belong to families of proteins that play roles in modulating host-pathogen interactions, usually by down-regulating plant defenses [52].

### M96 and prolyl 4-hydroxylase families display distinct trajectories of expansion

Studies focused on effectors such as RxLRs and CRNs have revealed that expansions and contractions within those families are responsible for many differences in gene content between species of *Phytophthora* [53]. To extend analyses to other gene families, we examined the M96 and prolyl 4-hydroxylase genes, many of which were mating-induced. This employed the genome sequences of eleven species of *Phytophthora*.

**Fig 3:**
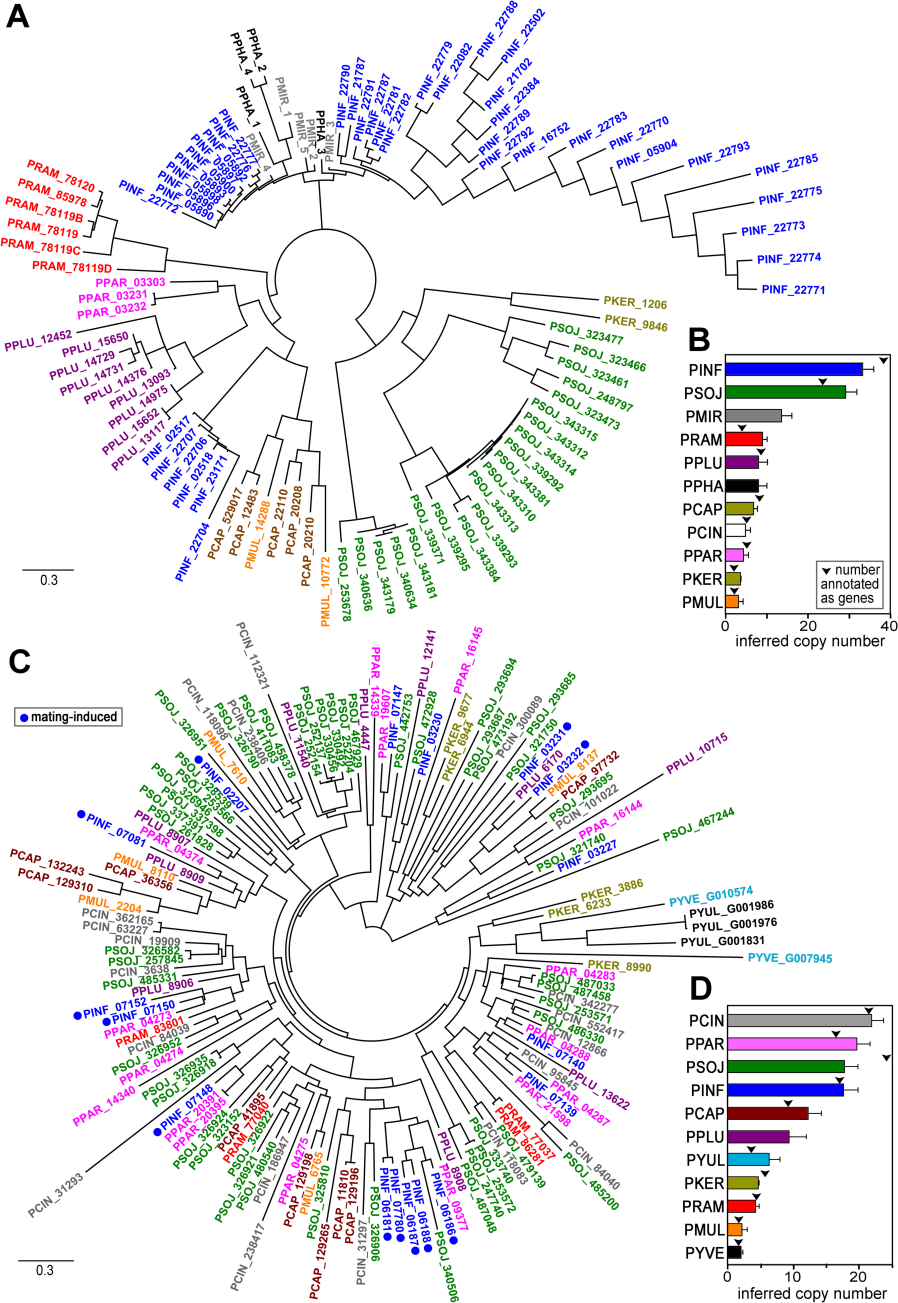
Evolution of two mating-induced gene families. **(A)** Maximum likelihood phylogenetic tree of M96 proteins from eleven species of *Phytophthora*. The species are color-coded and denoted by gene prefixes, which are *P*. *capsici* (PCAP), *P*. *cinnamomi* (PCIN), *P*. *infestans* (PINF), *P*. *kernoviae* (PKER), *P*. *mirabilis* (PMIR), *P*. *multivora* (PMUL), *P*. *parasitica* (PPAR), *P*. *phaseoli* (PPHA), *P*. *pluvalis* (PPLU), *P*. *ramorum* (PRAM), and *P*. *sojae* (PSOJ). **(B)** Copy number estimates of M96 genes based on hits in Illumina libraries of genomic DNA (bars). Arrowheads denote the number of loci annotated as genes in each species’ respective genome projects that have similarity to M96. **(C)** Prolyl hydroxylase (2OG) family in nine species of *Phytophthora* (labeled as in panels a and b) plus *Phytopythium vexans* (PYVE) and *Py*. *ultimum* (PYUL). **(D)** Number of prolyl hydroxylase gene copies calculated as described in panel B.

The size of the M96 family ranged from a low of two genes in *P*. *multivora* to more than 40 in *P*. *infestans*. In most species, the genes occur in one or more clusters of tandem repeats. A phylogenetic analysis of M96 proteins indicated that most form species-specific clusters (Fig 3A). This suggests either that a *Phytophthora* ancestor contained only a few M96 genes which later diverged and expanded in copy number, or that ancestral families underwent concerted evolution. A similar conclusion was drawn in our prior study, which was performed when only three sequenced *Phytophthora* genomes were available [22]. Although *Pythium* spp. encode proteins with similar amino acid characteristics such as signal peptides and glycine/serine-rich domains, none matched the *P*. *infestans* proteins based on an BlastP *E* cutoff of 10^−10^.

A few exceptions to the species-specific clustering of M96 proteins were observed. One involved proteins from *P*. *infestans*, *P*. *mirabilis*, and *P*. *phaseoli*, as the latter two were nested within a larger *P*. *infestans* clade. Prior studies have described the close similarity of the three species [54]. In addition, the *P*. *infestans* sequences formed two clusters, one grouping with *P*. *mirabilis* and *P*. *phaseoli* and the other with *P*. *capsici*. We also observed intermingling of M96 sequences from *P*. *capsici* and *P*. *multivora*, which are known to have close taxonomic affinity [55].

We considered, but later excluded, the possibility that the broad divergence in M96 family size was an artifact of errors in genome assembly or annotation. This was a concern since the initial Sanger-based assemblies of the *P*. *infestans* genome erroneously included only one M96 gene, due to overcollapsed sequence repeats. We therefore independently estimated M96 copy numbers using Illumina DNA reads in Genbank, by comparing the number of reads matching M96 versus single-copy genes (Fig 3B). The resulting estimates of M96 copies (bars in the figure) were usually similar to the number of annotated genes (inverted triangles). A two-fold discrepancy was observed for *P*. *ramorum*, however. The number of annotated M96 genes in *P*. *mirabilis* and *P*. *phaseoli* is not shown in Fig 3B since they lack a genome assembly; their sequences were obtained by PCR using degenerate primers based on the *P*. *infestans* sequences.

The evolutionary history of the prolyl 4-hydroxylase gene family differs from that of M96 in that the prolyl 4-hydroxylase proteins did not form species-specific clades in phylogenetic analyses (Fig 3C). Nevertheless, as with M96, copy numbers varied substantially between species (Fig 3D). This conclusion held both when considering gene numbers annotated in the genome projects and using our own assessments of copy number based on Illumina read counts. In addition, M96 and prolyl 4-hydroxylase copy numbers were not correlated. For example, *P*. *multivora* contained relatively few members of both families compared to the other species, while *P*. *cinnamomi* had the most genes encoding prolyl 4-hydroxylases but few M96 sequences. Interestingly, orthologs from *Pythium ultimum* and *Phytopythium vexans* (representing a genus intermediate to *Phytophthora* and *Pythium*) clustered with a subgroup of sequences from *P*. *kernoviae*.

### Self-fertiles express mating-induced genes at elevated levels

**Fig 4:**
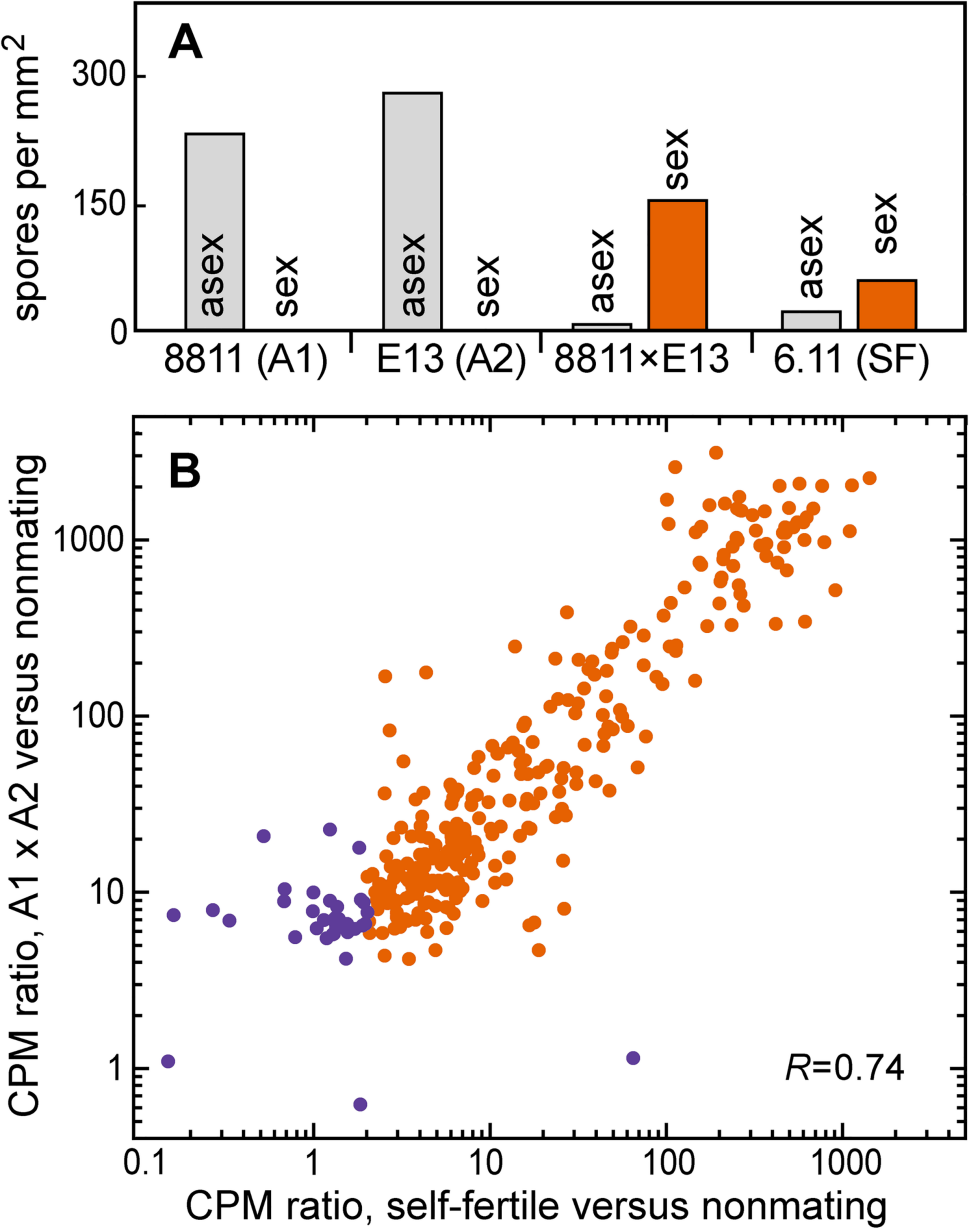
Analysis of self-fertile strain 6.11 of *P*. *infestans*. **(A)** Production of oospores and asexual spores by 6.11 compared to normal A1 and A2 strains (8811, E13) and a 8811 × E13 mating. **(B)** mRNA levels of the 455 mating-induced genes. The *x*-axis shows the CPM ratio of the genes in 6.11 compared to the nonmating controls, and the *y*-axis shows their ratio in the self-fertile strain compared to the nonmating controls. Orange symbols portray genes that are consistently elevated in expression in the oospore-forming cultures, based on 4- and 2-fold induction ratio thresholds in normal matings and in 6.11, respectively. Pearson's correlation coefficient (*r*) between the datasets was 0.74.

Most *P*. *infestans* strains are of the A1 or A2 mating type, but some natural isolates form oospores in single culture [56]. We previously isolated a self-fertile strain, 6.11, which may be a heterokaryon of A1 and A2 nuclei [15]. Oospore production by 6.11 is significant although not as high as in A1 × A2 matings (Fig 4A). Asexual sporulation is suppressed in 6.11, but not to the same extent as in normal matings.

About 85% of the genes induced >10-fold in at least one cross had elevated mRNA levels in strain 6.11, based on RNA-seq of two biological replicates (Fig 4B). Such genes were expressed at about three-fold lower levels in 6.11 than in the A1 × A2 matings, which is consistent with the observation that the self-fertile strain produced fewer oospores. Having higher mRNA levels in 6.11 were all of the major classes of genes described earlier. These included those encoding the M96 family, prolyl hydroxylases, homeodomain and pirin transcription regulators, glycosyltransferases, and tyrosinases. Most genes that were not transcribed at elevated levels in 6.11 lacked functional annotations.

### Detection of proteins in mature oospores

To identify proteins present in mature oospores, matings were allowed to proceed for 30 days. By this time, adjacent hyphae were highly vacuolated while oospores had formed thick inner walls within a thinner multilayered outer wall. Prior studies in *Phytophthora* have measured the thickness of the inner wall at about 3 μm [9].

**Fig 5:**
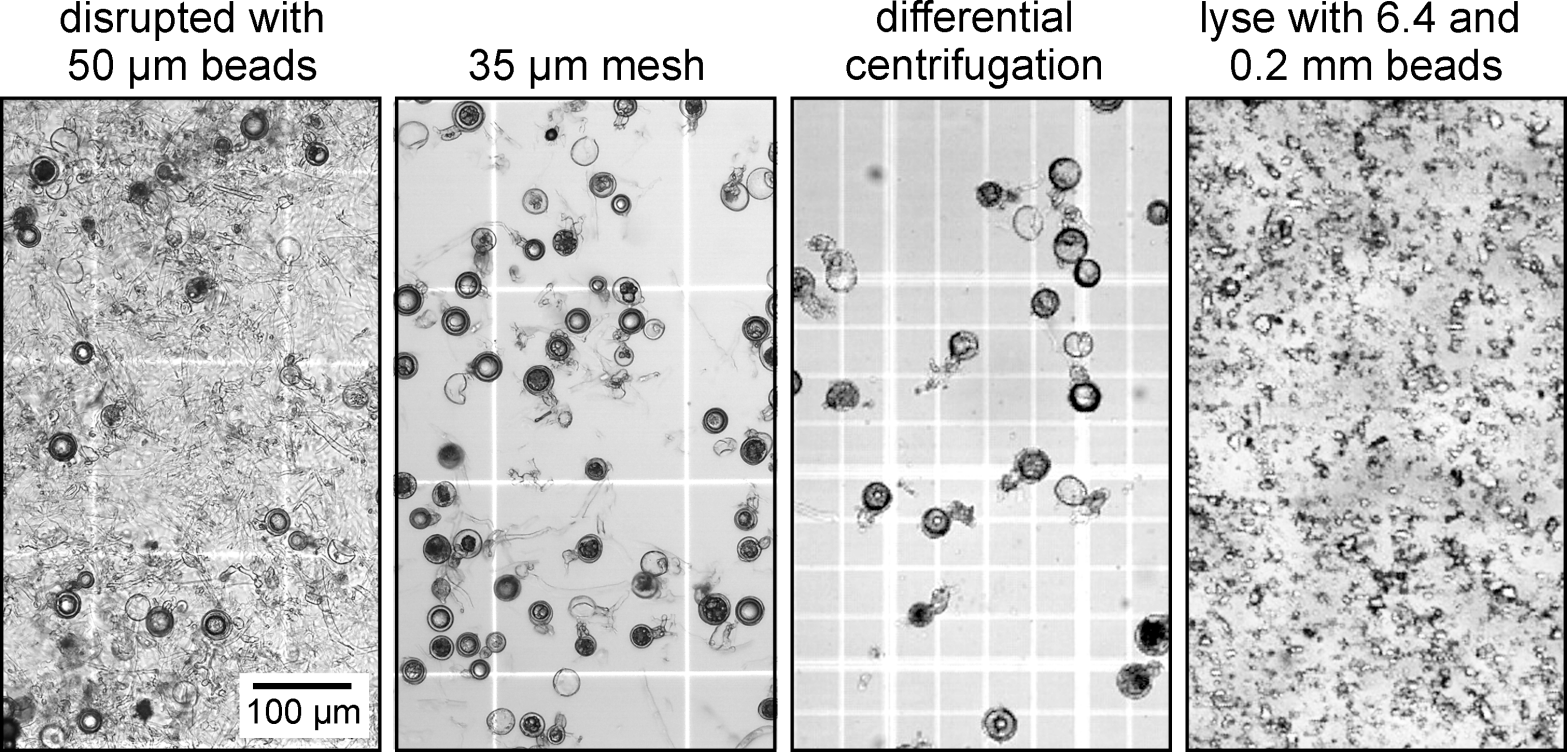
Processing of *P*. *infestans* mating zones for proteomics analysis. Indicated from left to right are the initial homogenate obtained by disrupting the sample with 50 μm beads, the sample after the final passage through nylon mesh, the sample after washing and differential centrifugation, and unclarified lysate obtained by shaking with 6 mm ceramic and 0.2 mm glass beads.

Both enzyme-based and mechanical methods for separating oospores from the hyphal mat for protein analysis were tested as described in Materials and Methods. The best results were obtained using the mechanical method that is illustrated in Fig 5. Tissue in the mating zones was initially disrupted by agitation with 50 μm glass beads. The resulting mixture was passed through 100 μm and 35 μm nylon mesh, which removed most hyphal fragments. The latter did not contain visible cytoplasm, but presumably still contained wall-associated proteins and were thus necessary to remove. This was achieved by differential centrifugation. The oospores were then broken by agitation with a mixture of ceramic and glass beads, and proteins solubilized in extraction buffer. Remaining cell wall fragments, including covalently bound and insoluble proteins, were removed by differential centrifugation.

**Fig 6:**
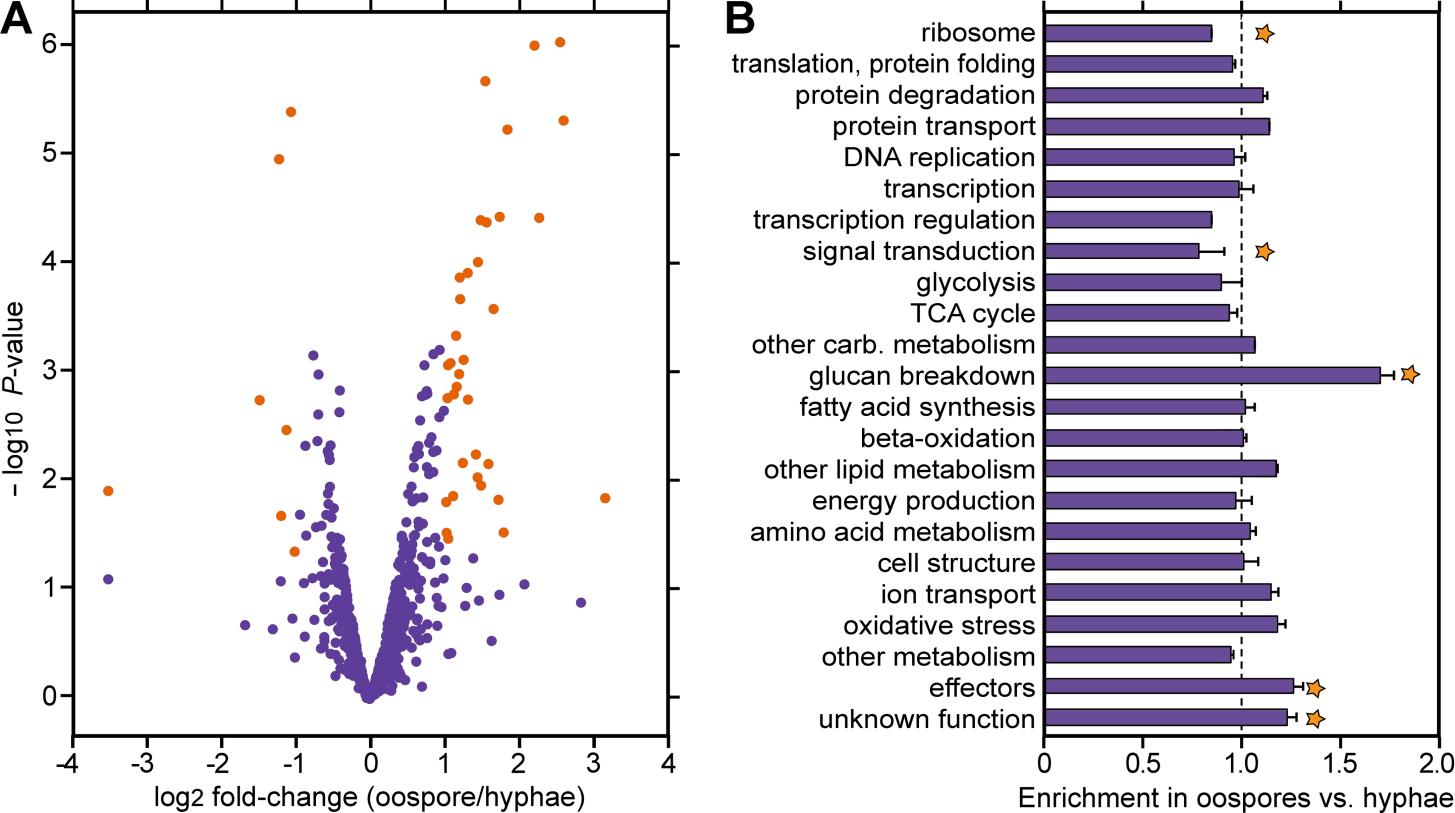
Analysis of proteins in oospores and vegetative hyphae. **(A)** Ratio of protein levels in oospores compared to hyphae based on iTraq analysis. Orange spots correspond to those that show >2-fold changes (log_2_ of 1) at *P*<0.05. **(B)** Enrichment of proteins in different functional classes. Values were determined by summing the median-normalized pseudospectral counts of all proteins in each functional class, and dividing the value for oospores by the sum from vegetative hyphae. Stars represent differences significant at *p*<0.05 based on Bayesian estimation, with error bars reflecting the biological replicates. Proteins in each category are shown in S6 Table.

Proteins from oospores resulting from the 8811 × 618 cross were compared to proteins from vegetative hyphae of the A1 and A2 parents by MudPIT [27]. To aid in quantification, we employed the iTRAQ method (isobaric tags for relative and absolute quantitation; [57]). For each biological replicate, about 200 mating plates were required to obtain enough oospore protein for analysis. A total of 839 proteins were identified from hyphae and 835 from oospores. As shown in Fig 6A, 37 proteins showed a two-fold or greater difference in abundance in oospores compared to non-mating cultures, based on a *p*-value threshold of 0.05. The proteins and their abundance are shown in S6 Table.

### Functions of proteins in mature oospores

Functions could be predicted for 760 of the proteins based on hits in GenBank and protein domain databases (S6 Table). Most common were proteins associated with metabolism. Of 263 such proteins, 85 were associated with the metabolism of carbohydrates, 56 with amino acids, 50 with fatty acid or lipids, and 30 with energy production through oxidative phosphorylation. The second largest group had functions related to protein synthesis. This included 66 ribosomal proteins, 100 involved in translation and protein folding, and 27 associated with protein trafficking.

A comparison of proteins in each functional class revealed a significant enrichment in oospores relative to vegetative hyphae of proteins participating in β-glucan degradation, such as β-1,3-glucanases (Fig 6B). These may be used to digest the oospore wall to allow germ tube emergence, or to liberate carbon for generating energy or metabolites. Effectors and proteins with unknown functions were also enriched in oospores. In contrast, ribosomal and signal transduction proteins were present at lower levels in oospores. The latter included protein kinases and GTP-binding proteins (S6 Table).

Despite prior reports that mitochondria in oospores are reduced in number or degraded [58], there was no difference in the abundance of mitochondrial proteins in oospores compared to hyphae. Predicted as mitochondrial proteins by the Mitofates and TargetP programs were 142 proteins. The ratio of their aggregate abundance in oospores compared to hyphae was 1.00, with 72 and 70 showing higher or lower levels in oospores, respectively. The only mitochondrial protein showing a >2-fold difference between oospores and hyphae was elongation factor-TS (PITG_17607), which was 2.1-fold more abundant in oospores.

One of our original goals was to characterize mRNA in mature oospores in addition to their proteins. However, we were unable to recover enough good-quality RNA to perform RNA-seq. Nevertheless, we compared levels of transcripts from the 10-day 8811 × 618 mating cultures with the corresponding proteins in the 30-day oospores from that pairing. As expected due to the differences between the tissues, the correlation was only modest, with Pearson's *r* = 0.27. A stronger correlation was seen when protein and RNA from vegetative hyphae were compared, with *r* = 0.42. This is within the range (0.4 to 0.6) reported for vegetative plant tissues [59].

### Identifying improved controls for gene expression studies

We took advantage of the RNA-seq data from this project plus our prior studies of asexual development and plant infection [33–35] to search for robust housekeeping gene controls. This involved comparing the expression stability of all *P*. *infestans* genes in 20 distinct growth conditions and life stages. These included matings, young and older asexual mycelia, mycelia grown on agar and in broth, mycelia grown on rich and defined media, asexual sporangia, zoospores, germinating zoospore cysts, fungicide-treated mycelia, and tomato leaves and potato tubers during biotrophic and necrotrophic (early and late) stages of infection.

**Fig 7:**
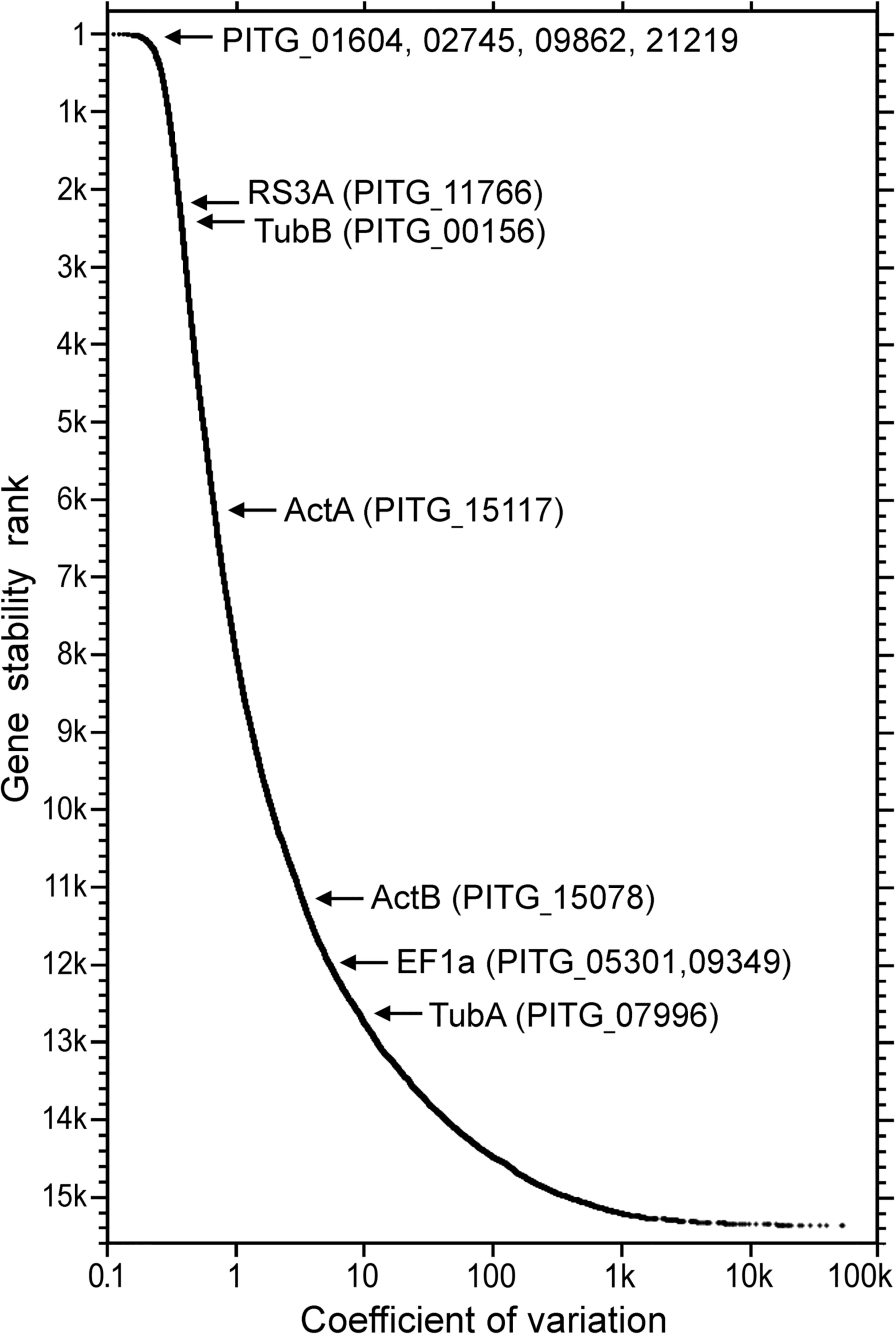
Expression stability of *P*. *infestans* genes based on 20 tissues analyzed by RNA-seq. Indicated by arrows are genes examined in this study (PITG_01604, 02745, 09862, 21219, 11766) and those used as housekeeping controls in prior studies of *Phytophthora* (ActA, ActB, EF1α, TubA, TubB, RS3A).

Most genes used by the *Phytophthora* community as controls for reverse transcription-polymerase chain reaction (RT-PCR) or RNA blot studies were found to exhibit unstable expression (Fig 7). These included genes for elongation factor 1α, actin A, and actin B. We previously used a more stable gene as a control, namely PITG_11766 which encodes ribosomal protein S3a (RS3A) [35]. Nevertheless, this gene ranked only in the 86th highest percentile of stability in the pooled RNA-seq data. The variability of standard controls used by the *Phytophthora* community indicated that additional controls are needed.

We therefore tested seven genes that ranked in the 99th percentile of stability using quantitative RT-PCR (RT-qPCR). Primers against three of the genes yielded high and often irreproducible Cq (quantification cycle) values in initial tests and were not studied further. More reliable amplifications were obtained using primers against genes PITG_01604 (serine palmitoyltransferase), PITG_02745 (casein kinase), PITG_09862 (Kelch domain protein) and PITG_21219 (nonsense-mediated mRNA decay protein). Their performance was tested against RNA from 21 developmental stages and growth conditions, using two biological replicates and a total of six technical replicates per sample type.

**Fig 8:**
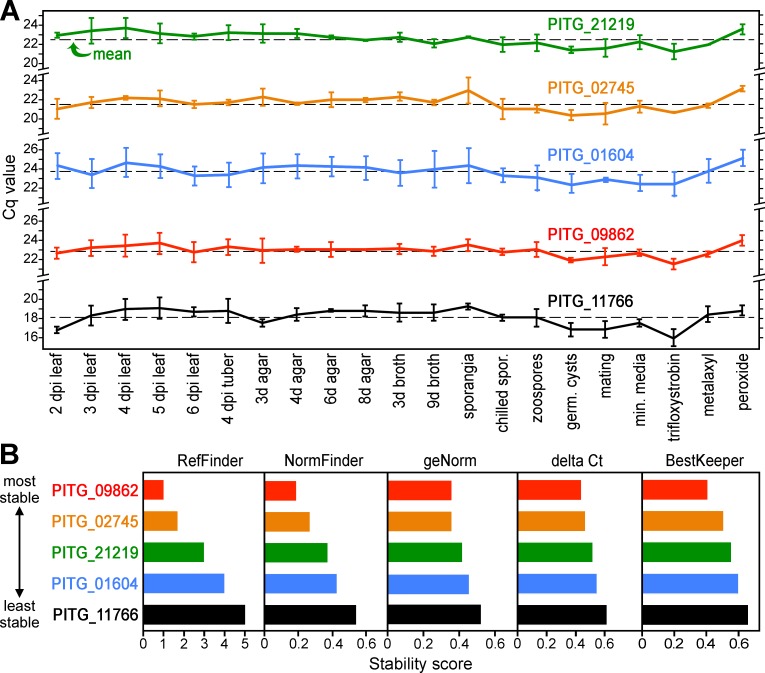
RT-qPCR of primer sets for five genes against 21 tissues. (**A)** Cq values. The dashed line represents the average for all samples, which are described in more detail in Materials and Methods. Each condition was represented by two biological replicates, each analyzed with three technical replicates. Error bars represent standard deviations. (**B)** ranking of performance of each primer set based on the Normfinder, geNorm, delta Ct, and BestKeeper algorithms, plus a comprehensive ranking based on the RefFinder program.

Each of the new genes outperformed PITG_11766 in terms of stability (Fig 8A). It should be noted that Cq values for infected plant tissues were adjusted based on the fraction of pathogen RNA, which was determined based on read-mapping statistics in RNA-seq analysis. The best overall results were yielded by PITG_09862 based on stability rankings provided by the NormFinder, geNorm, delta Ct, Bestkeeper, and RefFinder algorithms (Fig 8B). PITG_02745 was a close second. Interestingly, the most deviation was observed for samples treated with the toxins hydrogen peroxide and trifloxystrobin; the latter is a crop protection chemical that inhibits mitochondrial respiration in oomycetes and fungi.

### Validation of mating-induced genes by RT-qPCR

**Fig 9:**
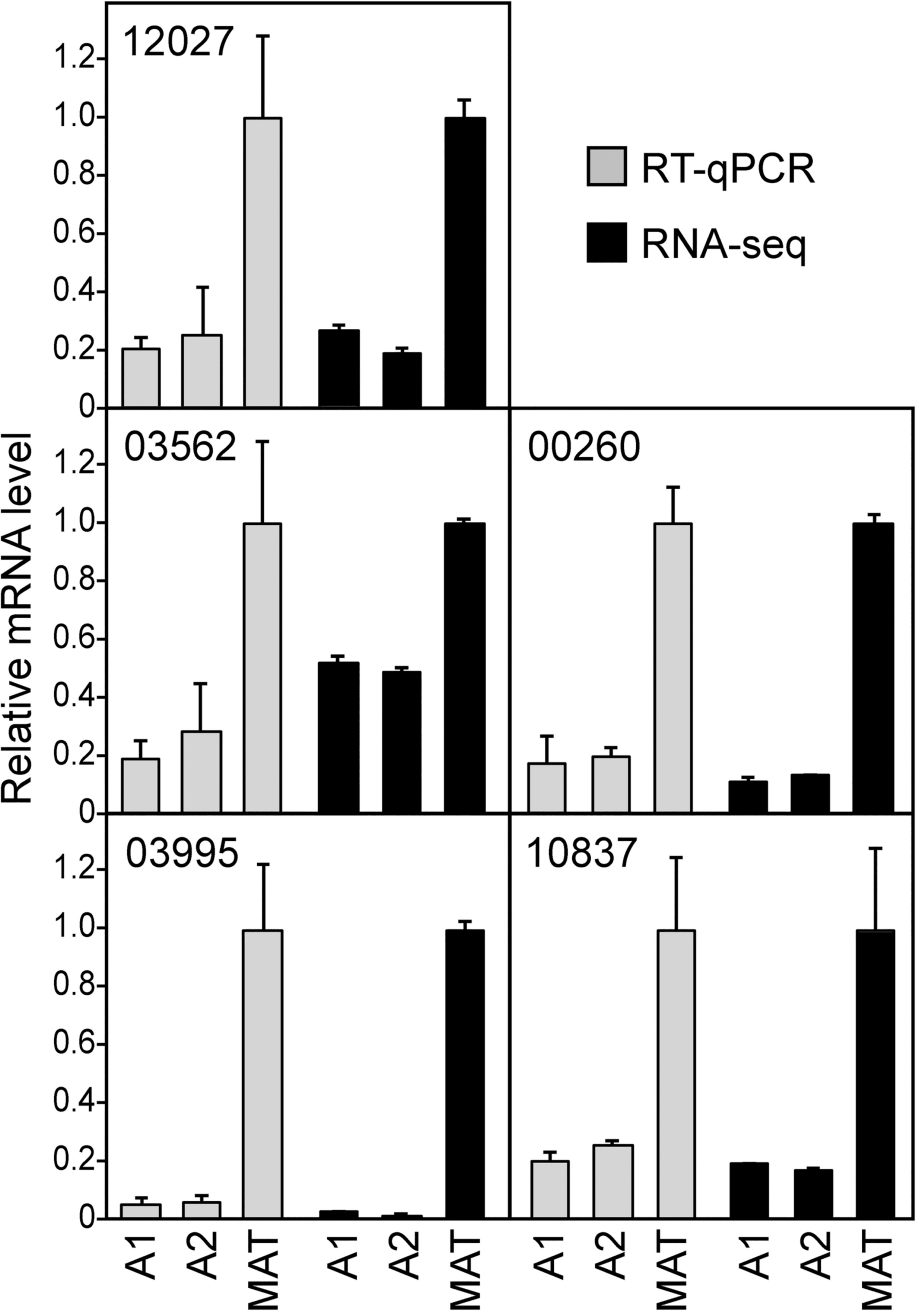
Validation of mating-induced genes by RT-qPCR. Grey bars illustrate results using primers against genes PITG_12027, 03562, 00260, 03995, and 10837 in strains 8811 (A1), E13 (A2) and a 8811 × E13 mating (MAT) using gene PITG_09862 as a housekeeping control. Black bars are the results from RNA-seq. The data are adjusted to show the mRNA levels in mating cultures as 1.0.

RT-qPCR was used to confirm that the genes identified by RNA-seq were induced during mating. This used our new housekeeping gene, PITG_09862, as a control. These assays involved testing four genes that increased modestly during mating (two to five-fold) and one gene that was induced strongly (about 20-fold). As shown in Fig 9, the results from RNA-seq and RT-qPCR were very consistent. There was some quantitative variation in the case of PITG_03562, however. While this gene was induced three-fold during mating based on RT-qPCR, RNA-seq indicated only two-fold induction.

### Total mRNA levels vary in different tissues

A lack of perfect concordance between relative gene expression levels measured by RT-qPCR and RNA-seq have been noted in this study and in other organisms [60]. To test one factor that might influence transcript measurements, we quantified the fraction of polyadenylated RNA in several life stages and in hyphae grown under different conditions. This involved two cycles of purification using oligo-dT beads, which were effective at removing rRNA based on purity assessments using an Agilent Bioanalyzer.

**Fig 10:**
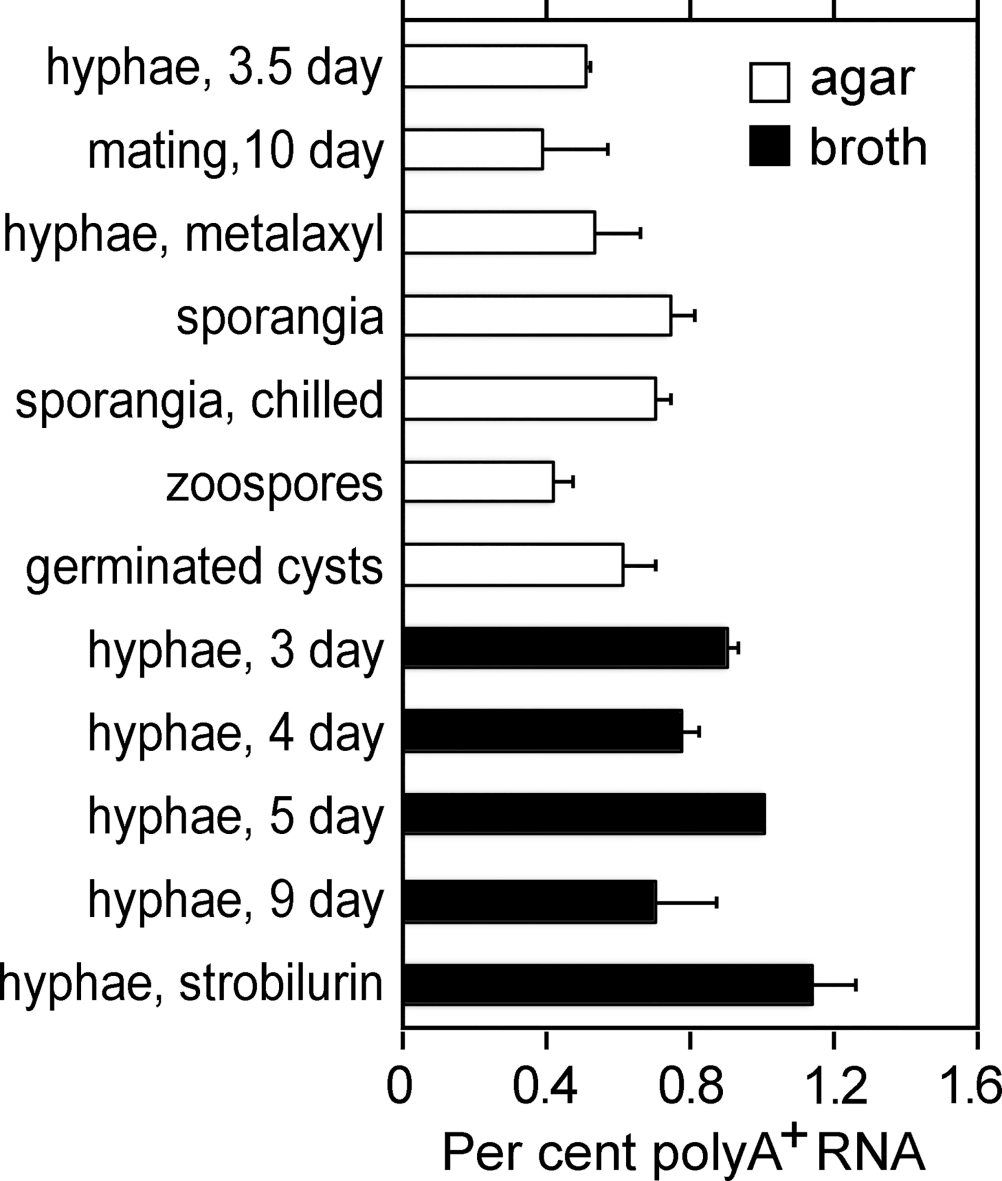
Polyadenylated RNA fraction of *P*. *infestans* tissues. Values are based on two biological replicates, using an oligo-dT binding assay.

The average polyA^+^ fraction was about 0.8% of total RNA, but this varied with developmental stage and growth condition (Fig 10). The differences were generally consistent between biological replicates and technical replicates that were assayed on different days. The polyA^+^ fraction was slightly higher in sporangia than hyphae, and lower in zoospores than sporangia. Interestingly, the polyA^+^ fraction was about 60% higher in hyphae from broth cultures compared to agar media. Transcription may be stimulated in broth since hyphae are more in contact with nutrients.

## Discussion

Few studies have addressed molecular aspects of the development of sexual spores in oomycetes, in contrast to the more extensive literature on their asexual spores. Several genes influencing oospore formation have been identified in *Phytophthora*, but they also affect general growth and their impact on oosporogenesis may be secondary [61–63]. One exception comes from the oomycete *Py*. *oligandrum*, where a protein that decorates the oospore surface was identified [43]. This study thus contributes to our knowledge of oosporogenesis by discovering new mating-induced genes as well as illuminating the protein content of the oospore. We identified 455 genes induced >10-fold in the three crosses, which is approximately five times the number discovered in our prior array study. Since gametangia and oospores account for only a few percent of total tissue within mating zones, their degree of up-regulation within mating structures is undoubtedly much higher. A further improvement upon our earlier work was our use of data from a non-mating timecourse to demonstrate that nearly all of these genes were authentically induced during mating compared to hyphae, and not simply down-regulated in the nonmating controls.

Many genes induced during mating had predicted functions consistent with roles in cell walls or the extracellular matrix. These proteins included the products of the M96 family, prolyl hydroxylases, glycosyltransferases, a transglutaminase, and others. These may help form the oospore wall or generate other features associated with mating, such as extracellular substances that bind antheridia to oogonia. In *P*. *infestans*, antheridial initials form in response to mating hormones, and then attach to and penetrate the oogonial initials. Oogonial initials then swell and mature into oospores. It should be noted that while some of the putative wall-associated proteins described in this paper are mating-specific, such as M96, others such as the prolyl hydroxylases have paralogs that are expressed at other stages of growth.

A surprising observation was that nearly ten percent of the genes that were induced >10-fold during mating in all three crosses belonged to the RxLR or CRN protein families, which are known to suppress plant defenses during infection. It is possible that these genes are induced by a physiological condition that is common to mating and plant infection, or are stored in anticipation of plant infection after germination. Alternatively, the annotations of these genes might be incorrect, since the effectors are defined by short motifs that occur frequently by chance [52]. Of the 11 mating-induced RxLRs, six were expressed >2-fold higher in matings compared to their peak expression in plants, while four were expressed >2-fold higher in plants than in matings, based on our prior study of infected tubers [34].

Many genes induced in mating encode proteins with roles in signal transduction, such as protein phosphatases and transcription factors which may orchestrate gametangia or oospore development. Interestingly, a comparison of RNA-seq data from mating with data from our prior study of asexual spore development [33] identifies very few signal transduction genes as being induced during both processes. This suggests that the processes that generate sexual and asexual spores are largely independent. One exception is PITG_15600, which encodes a Nipped-B ortholog. In animals and fungi, Nipped-B participates in sister chromatin cohesion [51]. In *P*. *infestans*, this protein may be required for the nuclear divisions that occur during sporangia development and in oospores after fertilization, and/or during gametangial meiosis.

Another interesting contrast between asexual and sexual spore development concerns how oospores prepare for germination. Oospores normally germinate to form 'germ sporangia', which morphologically resemble asexual sporangia. This resemblance extends to the ability of the germ sporangium to release zoospores when placed in water. Genes encoding flagellar proteins (*e*.*g*. intraflagellar transport proteins, basal body proteins, and inner and outer-arm dyneins) are induced during asexual sporulation [33], prior to germination. However, none of these genes were induced during oosporogenesis, suggesting that they are not expressed until after oospores germinate.

Our comparisons of the protein content of mature oospores with vegetative hyphae identified several interesting differences such as increased levels of β-1,3-glucanases, which may be involved either in degradation of the oospore wall or laminarin during germination. Another important finding was that levels of proteins involved in most core cellular processes, such as glycolysis and transcription, were similar. Before this study, this could not be taken for granted due to the unusual nature of oospores. Mature oospores contain little functional cytoplasm, with most space external to nuclei taken up by neutral lipid globules [58]. Whether all proteins needed for germination are preformed or must be synthesized *de novo* in a reactivated cytoplasm is unclear [9]. One study indicated that chloramphenicol, which blocks mitochondrial protein synthesis, did not block germination although inhibitors of cytoplasmic protein synthesis did reduce germination [64]. Our results suggest that most proteins needed to initiate germination may already be present in mature oospores. Our observation that proteins involved in β-oxidation were not more abundant in oospores than hyphae may suggest that the abundant lipid reserves of oospores may be used more to form plasma and vesicular membranes than as an energy source. It would be useful to study the molecular changes that occur during germination, although this would be technically challenging since germination is asynchronous with many oospores never exiting dormancy [64].

Besides contributing to an increased understanding of sexual development, this paper has addressed general issues in gene expression analysis and the mRNA content of cells. We are aware of only one systematic study of controls for gene expression in an oomycete [65]. That report tested 18 genes from *P*. *parasitica*, and concluded that *RS3A* was fairly stable and well-suited for studies of pathogenesis. Nevertheless, in our studies with *P*. *infestans* its signal fell two-fold during the course of infection based on RT-qPCR and RNA-seq [35].

A philosophical issue in expression studies is whether to normalize against so-called housekeeping genes or on a per cell, total RNA, or total mRNA basis. Some RNA-seq tools adjust expression based on a subset of genes that do not change, *e*.*g*. TMM normalization as implemented in programs such as edgeR [66]. However, neither the RNA content or the polyadenylated fraction of RNA are constant in cells. In *P*. *infestans*, we observed >2-fold changes in the polyA^+^ fraction of RNA from different life stages and growth conditions. Studies in other organisms have reported up to six-fold differences [67, 68].

Changes in polyadenylation may be important to *P*. *infestans* biology and should be the subject of future investigations. It should be noted that our measurements of polyA^+^ may not distinguish between the absence of polyadenylation and tail shortening, which would reduce the efficiency of binding to oligo-dT. Alterations in tail length would affect any cDNA-based expression method that uses an oligo-dT primer, although this could be minimized by using oligo-dT with anchor bases. Tail length is known to vary with developmental stage and the cell cycle in other organisms [69, 70]. Short tails are thought to be related to reduced translational efficiency and increased mRNA decay, although whether this holds for all cell types is controversial [71]. Even though global polyadenylation did not change during mating in *P*. *infestans*, there may be specific targets for deadenylation since one mating-induced gene (PITG_00483, which was upregulated 7 to 15-fold) encodes a Pumilio RNA-binding protein, which in other taxa triggers the deadenylation of specific mRNAs [72].

## Supporting information

S1 Table Primers used for RT-qPCR. (DOCX)

S2 Table RNA-seq statistics for mating and nonmating controls. Indicated are the number of reads per sample and the fraction mapping to the genome.
(XLSX)

S3 Table CPM values for genes during mating in the three crosses. (XLSX)

S4 Table Differential expression calculations from RNA-seq. Indicated are the fold-change, *p*-value, and FDR statistics for mating and nonmating samples.
(XLSX)

S5 Table Functions of genes induced by >10-fold during mating. (XLSX)

S6 Table Proteins detected in oospores and nonmating hyphae. Indicated are the abundance level, functional annotation, and categorization of the proteins.
(XLSX)
