# Supplementary material for: Transcriptomic and proteomic analysis reveals wall-associated and glucan-degrading proteins with potential roles in Phytophthora infestans sexual spore development
Source: PLoS One. 2018 Jun 13;13(6):e0198186. doi: 10.1371/journal.pone.0198186 (PMC5999078; doi:10.1371/journal.pone.0198186)
Supplement: S1 Table — (DOCX) [file pone.0198186.s001.docx]

**S1 Table. Primer sets used in RT-PCR assays.**

| Primer Set | Forward and reverse primers (5' to 3') | purpose |
| --- | --- | --- |
| PITG_00260 | CAGCTACGTTGTCATTACTT, GCTAACCTCCCTACTTCGAA | confirm mating-induced gene  (Fig. 8) |
| PITG_00536 | CTTGGAGAAAGCCCGTCAAG, GTTCGTCCTTCTTCAGCGTC | RT-PCR control |
| PITG_00536B | AAGCTCCTTGATCCAGACGG, AGATTTCCCAAGCTTGCGTC | RT-PCR control |
| PITG_01604 | CTCTGCGTGACAACAGCAAT, GAGAGAACTCCCCGACCTTC | RT-PCR control  (Fig. 7) |
| PITG_02745 | GCACCTGTTTTTCCTGACCT, GGTTCTCGGATTTGTTGTGG | RT-PCR control (Fig. 7) |
| PITG_02745B | TCTACGCACCTTCCGAGTTT, CCAGGTGACGTTCAGGTTTT | RT-PCR control |
| PITG_03562 | GATCCCAGCTACTCGCCTCTATTTG, GTAAGGTGGTGCGGGGAACTTGGAC | confirm mating-induced gene (Fig. 8) |
| PITG_03995 | AGCAGTTCGAGACCAAGACGCAAGA, ATCATAGTCACGGGCAGAGTAGAAA | confirm mating-induced gene (Fig. 8) |
| PITG_08366 | TGTTCGCTTTCATCAAGCAC, ACATCCAGCCCTTGTTGTCT | RT-PCR control |
| PITG_09828 | GAGCGCATCTACGTCAAGTG, TAGTGCGTTTGGACTGCTTG | RT-PCR control |
| PITG_09828B | TTACGACCAGCACCTGAACA, TAGTGCGTTTGGACTGCTTG | RT-PCR control |
| PITG_09862 | ATTCGTGGGAGCACCTTGTA, ACTTCCCTCCACTCGTCAAG | RT-PCR control  (Fig. 7) |
| PITG_10837 | TTAAGTGACAAACAATGGCGAATGC, TACTCATCAATACAGCGACCGGGTT | confirm mating-induced gene (Fig. 8) |
| PITG_11766 | TAAGACGACGGACGGATAC, AGGCACAAACTTCAGGAATAG | RT-PCR control  (Fig. 7) |
| PITG_12027 | TGAAGAAGCTGAAGAAGGGCGAGAA, CGATACAAGAGCCGCCACGAAAATG | confirm mating-induced gene (Fig. 8) |
| PITG_19147B | TTAATTTTGAGGCGCAGGGG, CAGATCACGAACGTCTTGGG | RT-PCR control |
| PITG_21219 | CATGACCCCAAAATCTGCTT, CCTTTGGAGCCACACTTCTC | RT-PCR control (Fig. 7) |

Names of primer sets are based on their target genes (PITG prefix). Sets ending in "B" represent alternative primer pairs.
